# Mortality and Length of Stay Associated With Community-Acquired *Escherichia coli* and *Staphylococcus aureus* Bloodstream Infections: An Observational Study in 9 Low- and Middle-Income Countries

**DOI:** 10.1093/cid/ciag275

**Published:** 2026-06-02

**Authors:** Jill Hopkins, Olga Tosas-Auguet, Naomi Waithira, Chris Painter, Clare L Ling, Tamalee Roberts, Thyl Miliya, Noah Obeng-Nkrumah, Japheth A Opintan, Emmanuel P Abbeyquaye, Raph L Hamers, Yulia Rosa Saharman, Robert Sinto, Mulya Rahma Karyanti, R Fera Ibrahim, Samuel O Akech, Elizabeth A Ashley, Anousone Douangnouvong, Khamla Choumlivong, Nicholas A Feasey, Diana Kululanga, Samantha Lissauer, Abhilasha Karkey, Narayan Kunwar, Justice Enosetale Erakhaiwu, Iruka N Okeke, Ini Adebiyi, Olukemi A Adekanmbi, Abiodun B Oduola, Babatunde O Ogunbosi, Kehinde A Ojifinni, Olukemi O Tongo, Ifeoma A Ude, Aaron O Aboderin, Olusegun Adekanle, Adeyemi T Adeyemo, Sylvester S Edward, Ugowe Osagie, Hoa Thi Nguyen, Thach Ngoc Pham, Giang Van Tran, Hương Thị Lan Hoàng, Tùng Hữu Trịnh, H Rogier van Doorn, Paul Turner, Sue J Lee

**Affiliations:** Cambodia Oxford Medical Research Unit, Angkor Hospital for Children, Siem Reap, Cambodia; Centre for Tropical Medicine and Global Health, Nuffield Department of Medicine, University of Oxford, Oxford, United Kingdom; Antimicrobial Resistance Department, World Health Organization, Geneva, Switzerland; Centre for Tropical Medicine and Global Health, Nuffield Department of Medicine, University of Oxford, Oxford, United Kingdom; Mahidol-Oxford Tropical Medicine Research Unit, Faculty of Tropical Medicine, Mahidol University, Bangkok, Thailand; Centre for Tropical Medicine and Global Health, Nuffield Department of Medicine, University of Oxford, Oxford, United Kingdom; Mahidol-Oxford Tropical Medicine Research Unit, Faculty of Tropical Medicine, Mahidol University, Bangkok, Thailand; Lao-Oxford-Mahosot Hospital-Wellcome Trust Research Unit, Microbiology Laboratory, Mahosot Hospital, Vientiane, Lao PDR; Cambodia Oxford Medical Research Unit, Angkor Hospital for Children, Siem Reap, Cambodia; Centre for Tropical Medicine and Global Health, Nuffield Department of Medicine, University of Oxford, Oxford, United Kingdom; Centre for Tropical Medicine and Global Health, Nuffield Department of Medicine, University of Oxford, Oxford, United Kingdom; Lao-Oxford-Mahosot Hospital-Wellcome Trust Research Unit, Microbiology Laboratory, Mahosot Hospital, Vientiane, Lao PDR; Cambodia Oxford Medical Research Unit, Angkor Hospital for Children, Siem Reap, Cambodia; Department of Microbiology, School of Biomedical and Allied Health Sciences, University of Ghana, Accra, Ghana; Department of Medical Microbiology, University of Ghana Medical School, Accra, Ghana; Paediatric Division, 37 Military Hospital, Accra, Ghana; Centre for Tropical Medicine and Global Health, Nuffield Department of Medicine, University of Oxford, Oxford, United Kingdom; Faculty of Medicine Universitas Indonesia, Oxford University Clinical Research Unit Indonesia, Jakarta, Indonesia; Department of Microbiology, Faculty of Medicine Universitas Indonesia, Dr. Cipto Mangunkusumo National Hospital, Jakarta, Indonesia; Department of Internal Medicine, Pelni Hospital, Jakarta, Indonesia; Department of Internal Medicine, Pelni Hospital, Jakarta, Indonesia; Division of Tropical and Infectious Diseases, Department of Internal Medicine, Faculty of Medicine Universitas Indonesia, Dr. Cipto Mangunkusumo National Hospital, Jakarta, Indonesia; Department of Child Health, Faculty of Medicine Universitas Indonesia, Dr. Cipto Mangunkusumo National Hospital, Jakarta, Indonesia; Department of Microbiology, Faculty of Medicine Universitas Indonesia, Dr. Cipto Mangunkusumo National Hospital, Jakarta, Indonesia; Department of Microbiology, University of Indonesia Hospital, Jakarta, Indonesia; Health Services Unit, KEMRI Wellcome Trust Research Programme, Kilifi, Kenya; Centre for Tropical Medicine and Global Health, Nuffield Department of Medicine, University of Oxford, Oxford, United Kingdom; Lao-Oxford-Mahosot Hospital-Wellcome Trust Research Unit, Microbiology Laboratory, Mahosot Hospital, Vientiane, Lao PDR; Lao-Oxford-Mahosot Hospital-Wellcome Trust Research Unit, Microbiology Laboratory, Mahosot Hospital, Vientiane, Lao PDR; Setthathirath Hospital, Vientiane, Lao PDR; Malawi Liverpool Wellcome Programme, Kamuzu University of Health Sciences, Blantyre, Malawi; School of Medicine, University of St Andrews, St Andrews, United Kingdom; Department of Clinical Sciences, Liverpool School of Tropical Medicine, Liverpool, United Kingdom; Malawi Liverpool Wellcome Programme, Kamuzu University of Health Sciences, Blantyre, Malawi; Malawi Liverpool Wellcome Programme, Kamuzu University of Health Sciences, Blantyre, Malawi; Institute for Infection and Global Health, University of Liverpool, Liverpool, United Kingdom; Oxford University Clinical Research Unit Nepal, Lalitpur, Nepal; Oxford University Clinical Research Unit Nepal, Lalitpur, Nepal; Department of Medical Microbiology and Parasitology, College of Medicine, University of Ibadan, Ibadan, Nigeria; Department of Pharmaceutical Microbiology, Faculty of Pharmacy and Department of Medical Microbiology and Parasitology, College of Medicine, University of Ibadan, Ibadan, Nigeria; Department of Medical Microbiology and Parasitology, University College Hospital, Ibadan, Nigeria; Department of Medicine, College of Medicine, University of Ibadan, University College Hospital, Ibadan, Nigeria; Department of Medical Microbiology and Parasitology, University College Hospital, Ibadan, Nigeria; Department of Paediatrics, University College Hospital, Ibadan, Nigeria; Department of Paediatrics, University of Ibadan, Ibadan, Nigeria; Department of Emergency Medicine, University College Hospital, Ibadan, Nigeria; Department of Paediatrics, College of Medicine, University of Ibadan, Ibadan, Nigeria; Paediatric Infectious Diseases Unit, University College Hospital, Ibadan, Nigeria; College of Health Sciences, Obafemi Awolowo University, Ile-Ife, Nigeria; College of Health Sciences, Obafemi Awolowo University, Ile-Ife, Nigeria; College of Health Sciences, Obafemi Awolowo University, Ile-Ife, Nigeria; College of Health Sciences, Obafemi Awolowo University, Ile-Ife, Nigeria; Department of Paediatrics, Obafemi Awolowo University Teaching Hospitals Complex, Ile-Ife, Nigeria; Oxford University Clinical Research Unit Hanoi, Hanoi, Vietnam; Institute for Training and Research in Tropical Diseases, National Hospital of Tropical Diseases, Hanoi, Vietnam; Institute for Training and Research in Tropical Diseases, National Hospital of Tropical Diseases, Hanoi, Vietnam; Department of Infectious Diseases, Hanoi Medical University, Hanoi, Vietnam; Hue Central Hospital, Hue, Vietnam; Children's Hospital 2, Ho Chi Minh City, Vietnam; Centre for Tropical Medicine and Global Health, Nuffield Department of Medicine, University of Oxford, Oxford, United Kingdom; Oxford University Clinical Research Unit Hanoi, Hanoi, Vietnam; Cambodia Oxford Medical Research Unit, Angkor Hospital for Children, Siem Reap, Cambodia; Centre for Tropical Medicine and Global Health, Nuffield Department of Medicine, University of Oxford, Oxford, United Kingdom; Centre for Tropical Medicine and Global Health, Nuffield Department of Medicine, University of Oxford, Oxford, United Kingdom; Mahidol-Oxford Tropical Medicine Research Unit, Faculty of Tropical Medicine, Mahidol University, Bangkok, Thailand; Department of Infectious Diseases, The Alfred Hospital and School of Translational Medicine, Monash University, Melbourne, Australia

**Keywords:** attributable mortality, antimicrobial resistance, bloodstream infection, surveillance, LMIC

## Abstract

**Background:**

Antimicrobial resistance disproportionally affects low- and middle-income countries. We calculated the burden of resistant and susceptible *Escherichia coli* and *Staphylococcus aureus* bloodstream infections (BSIs) in ACORN2, a prospective clinical surveillance study in adults and children.

**Methods:**

Patients with culture-confirmed community-acquired susceptible and resistant *E. coli* (third-generation cephalosporin susceptible [3GC-S] and resistant [3GC-R]) and *S. aureus* (methicillin-resistant [MRSA] and methicillin-susceptible [MSSA]) BSIs were compared against blood culture–negative patients (controls). Cause-specific hazard ratios (HRs) were calculated for competing events of in-hospital mortality and discharge alive. Length of hospital stay (LOS) was calculated.

**Results:**

From 22 802 blood cultures taken, there were 329 *E. coli* (177 [53.8%] 3GC-R) and 363 *S. aureus* (132 [36.4%] MRSA) BSIs. In-hospital mortality was 8.4% for controls, 20.1% for *E. coli* (20.3% 3GC-R; 19.7% 3GC-S), and 13.1% for *S. aureus* (12.2% MRSA; 13.7% MSSA). 3GC-S but not 3GC-R *E. coli* BSI cases had higher adjusted hazard of death than controls (HRs, 1.73 [95% confidence interval {CI}, 1.20–2.49] and 1.38 [95% CI, .99–1.94]); risk of dying was the same for 3GC-S compared with 3GC-R infections (*P* = .882). Neither MSSA nor MRSA BSI patients were more likely to die than controls (HRs, 1.33 [95% CI, .91–1.93] and 1.00 [95% CI, .61–1.66]) nor MSSA versus MRSA (*P* = .891). The hazard of discharge alive for both resistant and susceptible *S. aureus* and *E. coli* decreased significantly when compared with controls. LOS was 2 (95% CI, 0–3) days and 1 (95% CI, −1 to 3) day longer for resistant compared with susceptible infections, for *E. coli* and *S. aureus*.

**Conclusions:**

Antimicrobial-resistant infections were not associated with increased mortality in ACORN2. Drug-resistant BSI had longer LOS compared with susceptible infections, but CIs encompassed 0 days.

## Teaser

Prospective clinical antimicrobial resistance (AMR) surveillance in Asia and Africa identified increased length of stay in patients with *Escherichia coli* and *Staphylococcus aureus* bloodstream infection, increased mortality in patients with *E. coli*, and no evidence of AMR-related mortality in either *E. coli* or *S. aureus*.

## Graphical Abstract

**Graphical Abstract:**
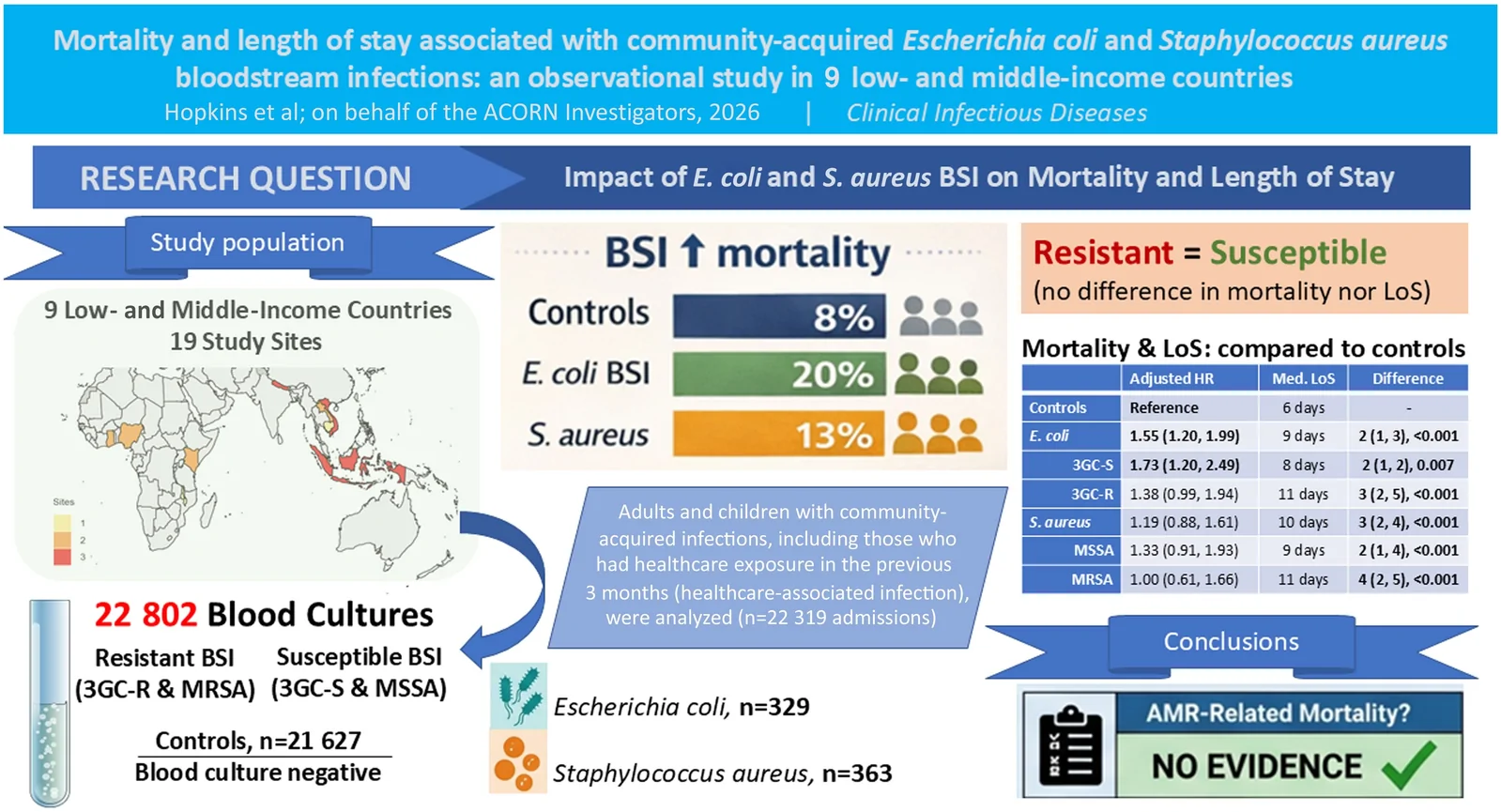

Antimicrobial resistance (AMR) is a global health threat and of particular concern in low- and middle-income countries (LMICs) where burden and impact are highest [1]. Quality surveillance data are a central component of response strategies, informing high-level action plans, as well as local treatment guidelines and infection prevention and control activities [2]. Most AMR surveillance data are from high-income settings and are often not linked with clinical data and outcomes, preventing accurate estimation of mortality attributable to infections and resistance [3–6]. Publications on the global AMR burden in 2019 and 2021 found that the lack of connected clinical and laboratory data, notably limited data on infection origin (ie, hospital-acquired infection [HAI] or community-acquired infection [CAI]), was a significant limitation in the generation of burden estimates [5, 7].

Bloodstream infections (BSIs) make up a large portion of the AMR burden [7, 8]. *Escherichia coli* and *Staphylococcus aureus* are the bacteria most frequently isolated from individuals with BSIs globally, and are priority pathogens in the World Health Organization (WHO) Global Antimicrobial Resistance and Use Surveillance System (GLASS) [9, 10]. *Escherichia coli* and *S. aureus* BSIs are frequently resistant to third-generation cephalosporin (3GC) antibiotics and methicillin, respectively. Resistant infections are thought to be associated with increased mortality, although evidence to support this in LMICs is scarce [11–14]. Notably, a recent study of *Enterobacterales* BSIs in Africa found no excess mortality attributable to 3GC resistance [11].

ACORN2 (A Clinically Oriented antimicrobial Resistance surveillance Network) is a prospective case-based surveillance project designed for incorporation into routine hospital workflow in LMICs [15]. ACORN2 uses active case finding and collects AMR-relevant clinical data, including outcomes, and associated microbiology laboratory data [16, 17]. The primary objective was to characterize drug-resistant infections by clinical syndrome, infection origin, patient age group, outcome, and geographic location [18]. For this substudy, additional data were analyzed from a subpopulation of all patients with blood culture taken to quantify the effects of *E. coli* and *S. aureus* BSIs on in-hospital mortality and length of hospital stay (LOS) for adults and children with CAIs.

## METHODS

### Study Design

ACORN2 was conducted in 19 sites in 9 countries in LMICs across Africa and Asia (Supplementary Figure 1 and Supplementary Table 1) from 7 September 2021 to 31 March 2024. All hospitalized patients of any age admitted to a participating ward with clinician-suspected acute bacterial infection and prescription of a parenteral antibiotic were eligible for enrollment. Patients with CAI were identified through daily surveillance of new admissions (≤48 hours postadmission). Any patient identified as having a CAI, but who had significant healthcare exposure in the previous 3 months, was classified as a healthcare-associated infection (HCAI). HCAIs and CAIs were combined for analysis.

Admitted patients who developed a clinically suspected infection >48 hours after admission, accompanied by initiation of a new parenteral antibiotic, were identified as HAI during weekly point prevalence surveys on surveillance wards. These patients were considered to have different etiology, represent a much smaller proportion of the surveillance population, and were analyzed separately.

All ACORN2 patients who were enrolled and had a blood culture taken but no microorganisms detected were assigned to the control group. This was in accordance with the WHO attributable mortality protocol, which states that controls “should consist of unexposed patients, which means patients without a BSI with one of the target pathogens at the moment of enrolment” [8]. In-hospital mortality was compared between patients with a resistant infection and no BSI and between patients with a susceptible infection and no BSI.

Ethical approval was granted by the Oxford Tropical Research Ethics Committee (OxTREC 524-21), with additional approvals from local/national ethics committees for each site. Full details of ACORN2 procedures are available at https://doi.org/10.6084/m9.figshare.27900042.v2. [15–17].

### Procedures

Upon enrollment, demographic, recent healthcare exposure, comorbidity, clinical syndrome and severity, and empiric antibiotic treatment data were recorded. When *E. coli* or *S. aureus* BSI was confirmed, additional clinical data were recorded, including immunosuppression, severity markers (Pitt bacteremia score variables) and treatment details according to the WHO attributable mortality protocol [8]. Upon hospital discharge, the patient's vital status was recorded. Microbiology data were extracted from local laboratory information management systems and merged with clinical data [19]. Processing and standardization of culture and antimicrobial susceptibility testing (AST) data are described elsewhere [18]. For this analysis, the AST category of “intermediate” (Clinical and Laboratory Standards Institute) or “susceptible, increased exposure” (European Committee on Antimicrobial Susceptibility Testing) was conservatively conflated with the category “susceptible” [20]. Methicillin resistance for *S. aureus* was determined using results from cefoxitin or oxacillin testing, and 3GC resistance (cefotaxime, ceftriaxone, or ceftazidime) for *E. coli*.

For severity scoring, quick Sequential Organ Failure Assessment (qSOFA) was used for adults, “Sepsis 6” for children, and WHO general danger signs for neonates [21, 22]. Neonates were aged ≤28 days, children were aged 29 days to <18 years, and patients ≥18 years of age were considered adults.

The primary outcomes for this analysis were hospital discharge status and hospital LOS. At discharge, patients who left against medical advice were categorized as alive and those who were “discharged to die at home” were combined with patients who died. Hospital LOS was defined as the number of days between admission and discharge or death.

### Statistical Analysis

As ACORN2 was a clinical AMR surveillance project that sought to enroll all eligible patients, no sample size was calculated. Analyses were done with Stata version 18.0 software [23].

Categorical variables were summarized using frequencies with percentage and continuous variables with medians and 25th and 75th percentiles (p25, p75). The number of severity markers were categorized into none, 1, or ≥2. Unadjusted comparisons between groups with BSIs and controls were made using a random-effects estimator to account for clustering by study site.

Cause-specific Cox proportional hazards regression models were used to calculate impact of target organism BSI on the competing events of discharge alive and in-hospital mortality. This allowed for assessment of the effect of BSI on in-hospital mortality while censoring for the competing event of discharge and vice versa. Because these were CAIs, there was no time-varying component; day of admission was used as time 0. The analysis was performed for all *E. coli* BSI admissions, regardless of antimicrobial susceptibility, and then separately for 3GC-resistant (3GC-R) and 3GC-susceptible (3GC-S) *E. coli* BSIs, using blood culture–negative admissions as controls. Similarly, separate models were run for all *S. aureus* BSIs and then for methicillin-resistant *S. aureus* (MRSA) and methicillin-susceptible *S. aureus* (MSSA) BSIs as exposures. Resistant infections were also compared against susceptible infections for both pathogens in models that did not include the controls. For admissions with >1 infection recorded, only the first *S. aureus* or *E. coli* BSI was considered.

Adjusted cause-specific models included age groups, elective or emergency hospital admission, sex (male or female), infection origin (CAI or HCAI), and the Charlson Comorbidity Index (CCI), unadjusted for age [24, 25]. Both unadjusted and adjusted models were stratified by study site to account for clustering and allow heterogeneity in baseline hazards [8]. For models comparing resistant with susceptible infections, empiric treatment appropriate for index infection was added as a covariate (Supplementary Figure 2). The proportional hazards assumption was assessed using Schoenfeld residuals and visually using log-log plots. Cox model results were expressed as hazard ratios (HRs) with 95% confidence intervals (CIs). These same procedures were repeated for analysis of HAI infections as a separate group.

For LOS, the number of days between admission and discharge or death was calculated and differences, plus 95% robust CIs, were calculated using the Hodges–Lehmann approach, adjusted for clustering by site using the between-cluster Somers’ D [26].

## RESULTS

**Figure 1:**
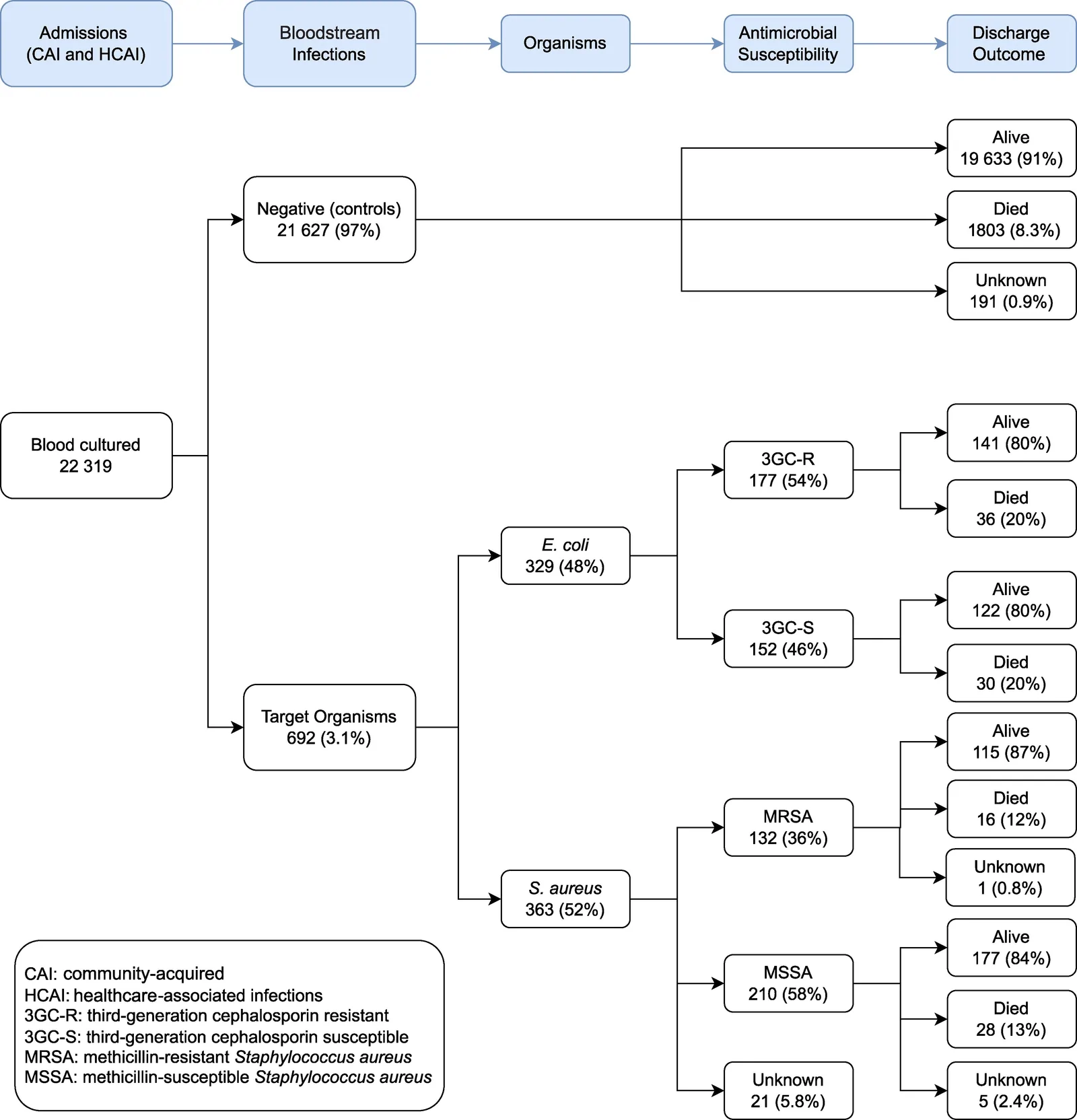
Participant flowchart showing enrolled surveillance participants with community-acquired infections and healthcare-associated infections who had blood cultures. Shown are numbers of bloodstream infection status, identified target organisms, antimicrobial resistance status, and discharge outcomes of alive, dead, or unknown.

There were 22 802 admissions with a laboratory-analyzed blood culture, including 17 221 (75.5%) CAIs and 5098 (22.4%) HCAIs (Table 1), plus 483 admissions with HAI (2.1%, described further in the Supplementary Results). Among CAI and HCAI BSIs, *E. coli* was detected in 329 admissions (1.5%), and *S. aureus* in 363 (1.6%). The remaining 21 627 (96.9%) infection episodes were culture negative and used as controls (Figure 1). There were no admissions with >1 *E. coli* or *S. aureus* infection or with both pathogens. Other pathogens detected are listed in Supplementary Table 2. There were 3 *S. aureus* isolates and 3 *E. coli* isolates that had intermediate susceptibility and were conflated into the susceptible group (Supplementary Results).

**Table 1.** Patient Characteristics for Community-Acquired Infection Groups and Controls

Characteristic	*Escherichia coli*	*Staphylococcus aureus*	Control	Total
Country of admission	329 (1.5%)	363 (1.6%)	21 627 (96.9%)	22 319 (100.0%)
Africa	83 (25.2%)	242 (66.7%)	10 194 (47.1%)	10 519 (47.1%)
Asia	246 (74.8%)	121 (33.3%)	11 433 (52.9%)	11 800 (52.9%)
Age, y, median (p25, p75)	51.0 (7.7, 69.0)	5.0 (0.1, 40.0)	4.5 (0.7, 38.1)	4.7 (0.7, 40.0)
Age category				
Neonate	26 (7.9%)	88 (24.2%)	3172 (14.7%)	3286 (14.7%)
Child	68 (20.7%)	156 (43.0%)	11 180 (51.7%)	11 404 (51.1%)
Adult	235 (71.4%)	119 (32.8%)	7275 (33.6%)	7629 (34.2%)
Age group				
<29 d	26 (7.9%)	88 (24.2%)	3172 (14.7%)	3286 (14.7%)
29 d to <1 y	30 (9.1%)	48 (13.2%)	3047 (14.1%)	3125 (14.0%)
1 to <5 y	19 (5.8%)	45 (12.4%)	4792 (22.2%)	4856 (21.8%)
5 to <18 y	19 (5.8%)	63 (17.4%)	3341 (15.5%)	3423 (15.3%)
18 to <55 y	84 (25.5%)	53 (14.6%)	3631 (16.8%)	3768 (16.9%)
≥55 y	151 (45.9%)	66 (18.2%)	3644 (16.9%)	3861 (17.3%)
Sex^a^				
Female	179 (54.4%)	143 (39.5%)	9431 (43.6%)	9753 (43.7%)
Male	150 (45.6%)	219 (60.5%)	12 183 (56.4%)	12 552 (56.3%)
Infection origin				
CAI	253 (76.9%)	291 (80.2%)	16 677 (77.1%)	17 221 (77.2%)
HCAI	76 (23.1%)	72 (19.8%)	4950 (22.9%)	5098 (22.8%)
Elective (vs emergency)^b^	25 (7.6%)	21 (5.8%)	969 (4.5%)	1015 (4.5%)
Median Pitt bacteremia score^a^ (p25, p75)	1.8 (2, 4)	2.1 (2, 9)	NA	2.0 (2, 6)
Pitt bacteremia score ≥4^a^	33 (14.2%)	34 (22.2%)	NA	67 (17.4%)
Median CCI score (p25, p75)	0.0 (0.0, 0.0)	0.0 (0.0, 0.0)	0.0 (0.0, 0.0)	0.0 (0.0, 0.0)
CCI score				
0	258 (78.4%)	305 (84.0%)	18 650 (86.2%)	19 213 (86.1%)
1	23 (7.0%)	27 (7.4%)	912 (4.2%)	962 (4.3%)
2	26 (7.9%)	22 (6.1%)	1497 (6.9%)	1545 (6.9%)
≥3	22 (6.7%)	9 (2.5%)	568 (2.6%)	599 (2.7%)
No. of severity markers				
0	100 (30.4%)	97 (26.7%)	7808 (36.1%)	8005 (35.9%)
1	109 (33.1%)	128 (35.3%)	6838 (31.6%)	7075 (31.7%)
≥2	107 (32.5%)	106 (29.2%)	5114 (23.6%)	5327 (23.9%)
Missing	13 (4.0%)	32 (8.8%)	1867 (8.6%)	1912 (8.6%)
Immunosuppression status 48 h prior to BC^a^	93 (35.6%)	53 (19.6%)	NA	146 (27.4%)
Empiric treatment appropriate for MSSA	182 (55.3%)	220 (60.6%)	12 981 (60.0%)	13 383 (60.0%)
Empiric treatment appropriate for MRSA	18 (5.5%)	27 (7.4%)	554 (2.6%)	599 (2.7%)
Empiric treatment appropriate for 3GC-S	286 (86.9%)	297 (81.8%)	18 245 (84.4%)	18 828 (84.4%)
Empiric treatment appropriate for 3GC-R	57 (17.3%)	31 (8.5%)	1410 (6.5%)	1498 (6.7%)
Empiric treatment appropriate for CPM-R^a^	0 (0.0%)	0 (0.0%)	36 (0.2%)	36 (0.2%)
Time from admission to discharge/death^a^, d, median (p25, p75)	9.0 (5.0, 14.0)	10.0 (5.0, 16.0)	6.0 (4.0, 10.0)	6.0 (4.0, 10.0)

Data are presented as No. (%) unless otherwise indicated. The breakdown by adult, child, and neonates is provided in Supplementary Table 3.

Abbreviations: 3GC-R, third-generation cephalosporin resistant; 3GC-S, third-generation cephalosporin susceptible; BC, blood culture; CAI, community-acquired infection; CCI, Charlson Comorbidity Index; CPM-R, carbapenem resistance; d, days; HCAI, healthcare-associated infection; MRSA, methicillin-resistant *Staphylococcus aureus*; MSSA, methicillin-susceptible *Staphylococcus aureus*; NA, not applicable; p25, 25th percentile; p75, 75th percentile.

^a^Sex unknown in n = 14 admissions; Pitt bacteremia score missing for n = 306 admissions (n = 96 *E. coli* and n = 210 *S. aureus*); immunosuppression status missing in n = 160 admissions (n = 68 *E. coli* and n = 92 S. *aureus*); and discharge date missing for n = 195 (n = 6 *S. aureus* and n = 189 controls).

^b^Elective defined as any planned admission and emergency defined as an unexpected or unplanned admission.

Among target organisms isolated from patients with BSIs at sites in Asia (n = 367), 67.0% were *E. coli*, whereas 74.5% of infections were *S. aureus* from sites in Africa (242/325) (Table 1 and Supplementary Table 3). The median (p25, p75) age for *E. coli* BSI was 51 years (8, 69 years) and 5 years both for *S. aureus* BSI (0.1, 40 years) and for controls (0.7, 38 years). Median (p25, p75) time from admission to discharge or death was 9 days for *E. coli* BSI (5, 14 days), 10 days for *S. aureus* BSI (5, 16 days), and 6 days for controls (4, 10 days).

Overall, there were 1913 in-hospital deaths (8.7%): 1803/21 436 among admissions with negative blood cultures (8.4%), significantly less than for admissions with *E. coli* (20.1% [66/329], *P* < .001) or *S. aureus* (13.1% [44/336], *P* = .005) (Table 2 and Supplementary Table 4). Among *E. coli* BSIs, 53.8% (177/329) were 3GC-R, and among *S. aureus* BSIs, 39.0% (131/336) were MRSA (Table 2). In-hospital mortality was not associated with resistance status in unadjusted analysis (with random effect for site) for either pathogen (*S. aureus*, *P* = .891; *E. coli*, *P* = .882). In-hospital mortality was 20% for both resistant and susceptible *E. coli* BSI admissions, which was significantly higher compared to controls (both *P* < .001), with a similar finding for MSSA (14%, *P* = .013). MRSA admissions were not associated with higher in-hospital mortality compared to controls (12.2%, *P* = .169).

**Table 2.** Outcome by Antimicrobial Susceptibility Testing Status for Community-Acquired Bloodstream Infections

Organism	Total	Alive	Died	*P* Value^c^	*P* Value^d^
No.^a^	22 101 (100.0%)	20 188 (91.3%)	1913 (8.66%)		
Controls	21 436 (100.0%)	19 633 (91.6%)	1803 (8.41%)		
*Escherichia coli*	329 (100.0%)	263 (79.9%)	66 (20.1%)		<.001
3GC-S	152 (46.2%)	122 (80.3%)	30 (19.7%)	.882	<.001
3GC-R	177 (53.8%)	141 (79.7%)	36 (20.3%)		<.001
*Staphylococcus aureus* ^b^	336 (100.0%)	292 (86.9%)	44 (13.1%)		.005
MSSA	205 (61.0%)	177 (86.3%)	28 (13.7%)	.891	.013
MRSA	131 (39.0%)	115 (87.8%)	16 (12.2%)		.169

Data are presented as No. (%) unless otherwise indicated. The breakdown by adult, child, and neonates is provided in Supplementary Table 4.

Abbreviations: 3GC-R, third-generation cephalosporin resistant; 3GC-S, third-generation cephalosporin susceptible; MRSA, methicillin-resistant *Staphylococcus aureus*; MSSA, methicillin-susceptible *Staphylococcus aureus*.

^a^n = 191 controls and n = 6 *S. aureus* admissions missing outcomewere excluded.

^b^n = 21 *S. aureus* admissions missing antimicrobial susceptibility testing result were excluded.

^c^Resistant compared with susceptible bloodstream infections.

^d^Compared with controls.

When adjusted, admissions with *E. coli* BSI had a higher hazard of in-hospital mortality compared to control admissions without BSI (adjusted HR [aHR], 1.55 [95% CI, 1.20–1.99]) (Table 3). Similarly, susceptible *E. coli* BSI (3GC-S) admissions were also more likely to die compared to controls (aHR, 1.73 [95% CI, 1.20–2.49]). Although the hazard of in-hospital mortality was also higher for 3GC-R *E. coli* BSI admissions compared to controls, this was not statistically significant (aHR, 1.38 [95% CI, .99–1.94]). There was a significant reduction in the hazard of discharge alive for both resistant and susceptible *E. coli* compared to controls, indicating a higher cumulative risk of in-hospital mortality. Similar to the unadjusted results, when 3GC-R *E. coli* BSIs were compared with 3GC-S *E. coli* BSIs, there was no difference in the risk of in-hospital mortality (aHR, 1.47 [95% CI, .57–3.79]) (Supplementary Table 6).

**Table 3.** Impact of *Escherichia coli* and *Staphylococcus aureus* Bloodstream Infection on Outcome When Compared With Controls

Bacteria	In-Hospital Mortality, HR (95% CI)		Discharged Alive, HR (95% CI)	
	Unadjusted	Adjusted	Unadjusted	Adjusted
*Escherichia coli*	1.82 (1.42–2.34)	1.55 (1.20–1.99)	0.69 (.61–.78)	0.72 (.64–.81)
	n = 21 765	n = 21 742	n = 21 765	n = 21 742
3GC-S	2.12 (1.47–3.05)	1.73 (1.20–2.49)	0.72 (.60–.86)	0.74 (.62–.89)
	n = 21 588	n = 21 565	n = 21 588	n = 21 565
3GC-R	1.60 (1.15–2.24)	1.38 (.99–1.94)	0.66 (.56–.79)	0.70 (.59–.83)
	n = 21 613	n = 21 590	n = 21 613	n = 21 590
*Staphylococcus aureus*	1.19 (.88–1.61)	1.19 (.88–1.61)	0.67 (.60–.76)	0.67 (.59–.75)
	n = 21 772	n = 21 748	n = 21 772	n = 21 748
MSSA	1.26 (.87–1.84)	1.33 (.91–1.93)	0.67 (.57–.77)	0.65 (.56–.75)
	n = 21 641	n = 21 618	n = 21 641	n = 21 618
MRSA	1.07 (.65–1.77)	1.00 (.61–1.66)	0.69 (.58–.84)	0.71 (.59–.85)
	n = 21 567	n = 21 543	n = 21 567	n = 21 543

Cause-specific HRs for competing events of discharge alive and in-hospital mortality. The n values indicate number of admissions included in the model. Both unadjusted and adjusted models were stratified by study site to account for clustering. Adjusted models included age groups (<29 days, 29 days to <1 year, 1 to <5 years, 5 to <18 years, 18 to <55 years, ≥55 years), sex (male or female), elective or emergency admission, Charlson Comorbidity Index (continuous), and infection origin (community-acquired infection or healthcare-associated infection).

Abbreviations: 3GC-R, third-generation cephalosporin resistant; 3GC-S, third-generation cephalosporin susceptible; CI, confidence interval; HR, hazard ratio; MRSA, methicillin-resistant *Staphylococcus aureus*; MSSA, methicillin-susceptible *Staphylococcus aureus*.

*Staphylococcus aureus* admissions and admissions with no BSI had similar risk of mortality in an adjusted model (aHR, 1.19 [95% CI, .88–1.61]) (Table 3). There was also no difference in the adjusted hazard of in-hospital mortality for MSSA BSI admissions nor MRSA BSI admissions compared to controls (Table 3). Similarly, when MSSA was compared with MRSA, the hazard of in-hospital mortality was not significantly different (aHR, 0.97 [95% CI, .38–2.52]) (Supplementary Table 6). There was a significant decrease in the adjusted hazard of discharge alive for both resistant and susceptible *S. aureus* BSI admissions when compared with controls (Table 3).

Results for HAI admissions (Supplementary Figure 3) are provided in Supplementary Table 5a (descriptives), Supplementary Table 5b (by outcome), and Supplementary Table 5c (cause-specific HRs).

For both *E. coli* and *S. aureus* BSIs, the unadjusted median LOS (p25, p75) was longer for resistant infections than for controls (11 days [5, 16 days] for 3GC-R *E. coli* and 11 days [6, 19 days] for MRSA vs 6 days [4, 10 days], both *P* < .001; Table 4). Median LOS was also longer for susceptible infections compared to controls (both *P* < .01; Table 4). The median difference ranged between 2 and 4 days longer for admissions with infection compared to controls. On average, 3GC-R *E. coli* admissions were longer by 2 days when compared with 3GC-S *E. coli* (95% CI, 0–3 days; *P* = .149) and MRSA admissions were 1 day longer when compared with MSSA (95% CI −1 to 3 days; *P* = .176).

**Table 4.** Length of Hospital Stay for Community-Acquired Bloodstream Infections

Group	No. (%)	Median LOS, d	p25, p75	Min, Max	Difference (95% CI), *P* Value^a^	Difference (95% CI), *P* Value^b^
Controls	21 436	6	4, 10	1, 492		
*Escherichia coli*	329	9	5, 14			2 (1–3), <.001
3GC-R	177 (53.8%)	11	5, 16	1, 58	2 (0–3), .149	3 (2–5), <.001
3GC-S	152 (46.2%)	8	5, 13	1, 61		2 (1–2), .007
*Staphylococcus aureus*	336	10	5, 16			3 (2–4), <.001
MRSA	131 (40.0%)	11	6, 19	1, 62	1 (−1, 3), .176	4 (2, 5), <.001
MSSA	205 (61.0%)	9	5, 15	1, 82		2 (1, 4), <.001

Median differences (95% CI) adjusted for clustering by study site.

Abbreviations: 3GC-R, third-generation cephalosporin resistant; 3GC-S, third-generation cephalosporin susceptible; CI, confidence interval; d, days; LOS, length of hospital stay; MRSA, methicillin-resistant *Staphylococcus aureus*; MSSA, methicillin-susceptible *Staphylococcus aureus*; p25, 25th percentile; p75, 75th percentile.

^a^Resistant compared with susceptible bloodstream infections.

^b^Compared with controls.

## DISCUSSION

In this prospectively recruited cohort of surveillance patients, mortality was 20% for both resistant and susceptible *E. coli* BSIs, 12%–14% for MSSA and MRSA BSIs, and 8% in blood culture–negative controls. When resistant infections were compared with susceptible infections, there was no significant difference in the hazard of in-hospital mortality for either pathogen. However, there was a significant decrease in the hazard of being discharged alive for patients with resistant and susceptible *S. aureus* and *E. coli* BSIs, translating to a longer in-hospital stay compared to controls. The average LOS was 2 days (95% CI, 0–3 days) and 1 day (95% CI, −1 to 3 days) longer for resistant compared with susceptible infections, for *E. coli* and *S. aureus*, respectively. Although not statistically significant, this difference may reflect whether the empiric treatment started upon admission was appropriate.

This study had a combined 308 resistant BSI admissions, which represented 46.5% of all *E. coli* and *S. aureus* infections. This is higher than in high-income countries (HICs), where a recent meta-analysis reported a prevalence of 36.5% (95% CI, 29%–44%) for AMR infections [27]. However, the proportion of deaths observed among *S. aureus* BSI admission was lower in this study when compared with HIC data, while mortality related to *E. coli* BSI fell within the range found in a study of 30-day mortality following infection by *E. coli* or *S. aureus* [28].

For *E. coli*, our results are similar to those reported by the MBIRA study (2020–2022) in 8 African hospitals, where patients with *Enterobacterales* BSIs had increased mortality compared to patients with no infection (HR, 6.79 [95% CI, 4.06–11.37]), but without differences in in-hospital deaths comparing 3GC-R and 3GC-S infections (HR, 0.74 [95% CI, .42–11.30]) [11]. An Indonesian study conducted at a single tertiary center (2015–2018) also found no excess mortality among 443 resistant versus 316 susceptible BSIs [29], albeit Indonesia may be considered an upper-middle-income country. In contrast, a meta-analysis of studies from Latin America (2000–2022) reported a significantly higher unadjusted odds of mortality among resistant infections (1.86 [95% CI, 1.55–2.23]), but also identified a downward trend of this risk over time [28, 30].

A large multicountry European BSI study (TIMBER, 2010–2011) identified increased in-hospital mortality in 3GC-R versus 3GC-S Enterobacteriaceae infections (HR, 1.39 [95% CI, 1.02–1.90]; n = 2560), but not in MRSA compared with MSSA infections (HR, 1.19 [95% CI, .81–1.75]; n = 1048) [31]. Furthermore, a meta-analysis of European studies identified increased mortality in resistant versus susceptible infections for dominant BSI organisms, highlighting the heterogeneity of mortality findings among studies [31]. Most papers included in the latter meta-analysis did not adjust for confounders such as infection origin, although 1 subgroup analysis did find higher hospital-acquired mortality for MRSA versus MSSA.

Differences between patient populations, health-seeking behaviors, antimicrobial use, empiric therapy, and provision of supportive care may explain some of these mortality differences among and between HIC and LMIC settings, but further LMIC studies are required to confirm these findings.

There were several potential limitations to this implementation of the WHO attributable mortality protocol within ACORN2. First, because of the small numbers of patients with HAI, primary analyses were focused only on CAI, while adjusting for recent healthcare exposure. The WHO protocol aims to recruit both CAI and HAI, with specific focus on HAI in the analysis plan [8]. As both mortality and resistance were higher among HAI versus CAI in ACORN2, further studies on HAI in LMICs are needed. Second, the WHO protocol states that both patients not diagnosed with suspected BSI, and therefore not blood sampled, or patients with confirmed BSIs by other pathogens or other type of infections can be used as a control group for comparison [8]. Consistent with this definition, this study used patients who were enrolled because of suspicion of infection but who had a negative blood culture as controls. Thus, we were able to adjust for confounders relevant to our settings using >17 000 blood culture–negative controls in cause-specific models. Within our control group, however, some participants with fever and markers of a severe inflammatory response syndrome were included, and there will have been individuals with inadequately treated infections and/or those who likely would satisfy a pragmatic definition of sepsis. These patients will have contributed to a higher risk of death than in ideal controls, that is, people from the community without signs or symptoms of infection, and this will have diminished differences between groups. Third, the WHO protocol specifies a large sample size of positive blood cultures for single settings. Even in this 19-site study lasting for >2 years, recruiting >40 000 patients receiving intravenous antibiotics, with >60% of infections blood cultured and aggregated resistance percentages of 50% and 35% for *E. coli* or *S. aureus* [18], we were unable to reach this target sample size to have 0.8 power to detect a 1.7 HR with 0.05 significance [8]. Larger or more selective studies, such as the newly developed isolate-initiated ACORN-lite protocol, may be able to achieve optimal sample sizes [32]. National surveys of AMR prevalence and mortality building on WHO standards [8, 33] are complementary and can also help increase the numbers of data points.

There were also intrinsic limitations to the ACORN2 protocol and network including challenges regarding obtaining some of the clinical variables (notably details of immunosuppression and the elements of the Pitt bacteremia score).

Two-thirds of patients recruited into ACORN2 were aged <18 years, which may explain a lower overall mortality than described in other studies. ACORN2 was hospital based and limited to higher level hospitals with clinical microbiology laboratories, assessment of attributable mortality was limited to symptomatic patients with access to these healthcare facilities, who were hospitalized there, had a blood culture done, and stayed to be assessed for vital status.

In this study we focused on CAI, including the subgroup of HCAI, because numbers for HAI were too small as a result of the pragmatic surveillance design. Given the differences in epidemiology, resistance, and mortality between community and hospital-origin infections, it is imperative for any future AMR surveillance and research to collect data on infection origin and to conduct stratified analyses taking this difference into account, including for excess mortality as also highlighted in a recent systematic review [34].

In conclusion, and consistent with results from the limited number of other LMIC-based studies, AMR BSIs were not associated with increased mortality compared to susceptible infections. Drug-resistant BSIs resulted in increased, but not statistically significant, LOS compared with susceptible infections. More importantly, we highlighted how, using the ACORN approach, we have been able to generate a large dataset of linked clinical, outcome, and laboratory data of drug-susceptible and -resistant infections in LMICs. These and future large clinically linked microbiology datasets are essential to directly inform future global modeling studies for more accurate real-world data–driven estimates of the global AMR burden.

## Supplementary Material

ciag275_Supplementary_Data

## Supplementary Data

Supplementary materials are available at *Clinical Infectious Diseases* online. Consisting of data provided by the authors to benefit the reader, the posted materials are not copyedited and are the sole responsibility of the authors, so questions or comments should be addressed to the corresponding author.
