# Supplementary material for: Mortality and Length of Stay Associated With Community-Acquired Escherichia coli and Staphylococcus aureus Bloodstream Infections: An Observational Study in 9 Low- and Middle-Income Countries
Source: Clin Infect Dis. 2026 Jun 2;83(2):e291–9. doi: 10.1093/cid/ciag275 (PMC13537153; doi:10.1093/cid/ciag275)
Supplement: Supplementary file 1 [file ciag275_supplementary_data.docx]

**ACORN Investigators**

| Author Number | Name | Affiliation | Contributions |
| --- | --- | --- | --- |
| 1 (first author) | Jill Hopkins | Cambodia Oxford Medical Research Unit, Angkor Hospital for Children, Siem Reap, Cambodia | Project administration; Validation; Formal analysis; Visualization; Writing—Original Draft |
|  |  | Centre for Tropical Medicine and Global Health, Nuffield Department of Medicine, University of Oxford, Oxford, UK |  |
| 2 | Olga Tosas Auguet | Surveillance, Prevention and Control Department, Antimicrobial Resistance Division, World Health Organization, Geneva, Switzerland | Methodology; Writing—Review & Editing |
| 3 | Naomi Waithira | Mahidol-Oxford Tropical Medicine Research Unit, Faculty of Tropical Medicine, Mahidol University, Bangkok, Thailand | Data curation; Writing—Review & Editing |
|  |  | Centre for Tropical Medicine and Global Health, Nuffield Department of Medicine, University of Oxford, Oxford, UK |  |
| 4 | Chris Painter | Mahidol-Oxford Tropical Medicine Research Unit, Faculty of Tropical Medicine, Mahidol University, Bangkok, Thailand | Formal analysis; Writing—Review & Editing |
|  |  | Lao-Oxford-Mahosot Hospital-Wellcome Trust Research Unit, Microbiology Laboratory, Mahosot Hospital, Vientiane, Lao PDR |  |
|  |  | Centre for Tropical Medicine and Global Health, Nuffield Department of Medicine, University of Oxford, Oxford, UK |  |
| 5 | Clare L. Ling | Cambodia Oxford Medical Research Unit, Angkor Hospital for Children, Siem Reap, Cambodia | Methodology; Validation; Writing—Review & Editing |
|  |  | Centre for Tropical Medicine and Global Health, Nuffield Department of Medicine, University of Oxford, Oxford, UK |  |
| 6 | Tamalee Roberts | Lao-Oxford-Mahosot Hospital-Wellcome Trust Research Unit, Microbiology Laboratory, Mahosot Hospital, Vientiane, Lao PDR | Methodology; Validation; Writing—Review & Editing |
|  |  | Centre for Tropical Medicine and Global Health, Nuffield Department of Medicine, University of Oxford, Oxford, UK |  |
| 7 | Thyl Miliya | Cambodia Oxford Medical Research Unit, Angkor Hospital for Children, Siem Reap, Cambodia | Investigation; Writing—Review & Editing |
| 8 | Noah Obeng-Nkrumah | Department of Microbiology, School of Biomedical and Allied Health Sciences, University of Ghana, Accra, Ghana | Investigation; Writing—Review & Editing |
| 9 | Japheth A. Opintan | Department of Medical Microbiology, University of Ghana Medical School, Accra, Ghana | Conceptualization; Supervision; Writing—Review & Editing |
| 10 | Emmanuel P.  Abbeyquaye | Paediatric Division, 37 Military Hospital, Accra, Ghana | Investigation; Writing—Review & Editing |
| 11 | Raph L. Hamers | Oxford University Clinical Research Unit Indonesia, Faculty of Medicine Universitas Indonesia, Jakarta, Indonesia | Conceptualization; Supervision; Writing—Review & Editing |
|  |  | Centre for Tropical Medicine and Global Health, Nuffield Department of Medicine, University of Oxford, Oxford, UK |  |
| 12 | Yulia Rosa Saharman | Department of Microbiology, Faculty of Medicine Universitas Indonesia, dr. Cipto Mangunkusumo National Hospital, Jakarta, Indonesia | Investigation; Writing—Review & Editing |
|  |  | Pelni Hospital, Jakarta, Indonesia |  |
| 13 | Robert Sinto | Division of Tropical and Infectious Diseases, Department of Internal Medicine, Faculty of Medicine Universitas Indonesia, dr. Cipto Mangunkusumo National Hospital, Jakarta, Indonesia | Investigation; Writing—Review & Editing |
|  |  | Pelni Hospital, Jakarta, Indonesia |  |
| 14 | Mulya Rahma Karyanti | Department of Child Health, Faculty of Medicine Universitas Indonesia, dr. Cipto Mangunkusumo National Hospital, Jakarta, Indonesia | Investigation; Writing—Review & Editing |
| 15 | R. Fera Ibrahim | Department of Microbiology, Faculty of Medicine Universitas Indonesia, dr. Cipto Mangunkusumo National Hospital, Jakarta, Indonesia | Investigation; Writing—Review & Editing |
|  |  | University of Indonesia Hospital, Jakarta, Indonesia |  |
| 16 | Samuel O. Akech | KEMRI Wellcome Trust Research Programme, Kilifi, Kenya | Conceptualization; Supervision; Writing—Review & Editing |
| 17 | Elizabeth A. Ashley | Lao-Oxford-Mahosot Hospital-Wellcome Trust Research Unit, Microbiology Laboratory, Mahosot Hospital, Vientiane, Lao PDR | Conceptualization; Supervision; Writing—Review & Editing |
|  |  | Centre for Tropical Medicine and Global Health, Nuffield Department of Medicine, University of Oxford, Oxford, UK |  |
| 18 | Anousone Douangnouvong | Lao-Oxford-Mahosot Hospital-Wellcome Trust Research Unit, Microbiology Laboratory, Mahosot Hospital, Vientiane, Lao PDR | Investigation; Writing—Review & Editing |
| 19 | Khamla Choumlivong | Setthathirath Hospital, Vientiane, Lao PDR | Investigation; Writing—Review & Editing |
| 20 | Nicholas A. Feasey | Malawi Liverpool Wellcome Programme, Kamuzu University of Health Sciences, Blantyre, Malawi | Conceptualization; Supervision; Writing—Review & Editing |
|  |  | School of Medicine, University of St Andrews, St Andrews, UK |  |
|  |  | Department of Clinical Sciences, Liverpool School of Tropical Medicine, Liverpool, UK |  |
| 21 | Diana Kululanga | Malawi Liverpool Wellcome Programme, Kamuzu University of Health Sciences, Blantyre, Malawi | Investigation; Writing—Review & Editing |
| 22 | Samantha Lissauer | Malawi Liverpool Wellcome Programme, Kamuzu University of Health Sciences, Blantyre, Malawi | Investigation; Writing—Review & Editing |
|  |  | Institute for Infection and Global Health, University of Liverpool, Liverpool, UK |  |
| 23 | Abhilasha Karkey | Oxford University Clinical Research Unit Nepal, Lalitpur, Nepal | Conceptualization; Supervision; Writing—Review & Editing |
| 24 | Narayan Kunwar | Oxford University Clinical Research Unit Nepal, Lalitpur, Nepal | Investigation; Writing—Review & Editing |
| 25 | Justice Enosetale Erakhaiwu | Department of Medical Microbiology and Parasitology, College of Medicine, University of Ibadan, Ibadan, Nigeria | Investigation; Writing—Review & Editing |
| 26 | Iruka N. Okeke | Department of Pharmaceutical Microbiology, Faculty of Pharmacy/Department of Medical Microbiology and Parasitology, College of Medicine, University of Ibadan, Ibadan, Nigeria | Conceptualization; Supervision; Writing—Review & Editing |
| 27 | Ini Adebiyi | Department of Medical Microbiology and Parasitology, University College Hospital, Ibadan, Nigeria | Investigation; Writing—Review & Editing |
| 28 | Olukemi A. Adekanmbi | Department of Medicine, College of Medicine, University of Ibadan, Ibadan, Nigeria | Investigation; Writing—Review & Editing |
| 29 | Abiodun B. Oduola | Department of Medical Microbiology and Parasitology, University College Hospital, Ibadan, Nigeria | Investigation; Writing—Review & Editing |
| 30 | Babatunde Oluwatosin Ogunbosi | University College Hospital, Ibadan, Nigeria | Investigation; Writing—Review & Editing |
|  |  | University of Ibadan, Ibadan, Nigeria |  |
| 31 | Kehinde Abraham Ojifinni | Department of Emergency Medicine, University College Hospital, Ibadan, Nigeria | Investigation; Writing—Review & Editing |
| 32 | Olukemi O. Tongo | Department of Paediatrics, College of Medicine, University of Ibadan, Ibadan, Nigeria | Investigation; Writing—Review & Editing |
| 33 | Ifeoma Azuka Ude | Paediatric Infectious Diseases Unit, University College Hospital, Ibadan, Nigeria | Investigation; Writing—Review & Editing |
| 34 | Aaron O. Aboderin | College of Health Sciences, Obafemi Awolowo University, Ile-Ife, Nigeria | Investigation; Writing—Review & Editing |
| 35 | Olusegun Adekanle | College of Health Sciences, Obafemi Awolowo University, Ile-Ife, Nigeria | Investigation; Writing—Review & Editing |
| 36 | Adeyemi T. Adeyemo | College of Health Sciences, Obafemi Awolowo University, Ile-Ife, Nigeria | Investigation; Writing—Review & Editing |
| 37 | Sylvester S. Edward | College of Health Sciences, Obafemi Awolowo University, Ile-Ife, Nigeria | Investigation; Writing—Review & Editing |
| 38 | Ugowe Osagie | Obafemi Awolowo University Teaching Hospitals Complex, Ile-Ife, Nigeria | Investigation; Writing—Review & Editing |
| 39 | Hoa Thi Nguyen | Oxford University Clinical Research Unit Hanoi, Hanoi, Vietnam | Investigation; Writing—Review & Editing |
| 40 | Thach Ngoc Pham | National Hospital of Tropical Diseases, Hanoi, Vietnam | Supervision; Project administration; Writing—Review & Editing |
| 41 | Giang Van Tran | National Hospital of Tropical Diseases, Hanoi, Vietnam | Supervision; Project administration; Writing—Review & Editing |
|  |  | Hanoi Medical University, Hanoi, Vietnam |  |
| 42 | Hương Thị Lan Hoàng | Hue Central Hospital, Hue, Vietnam | Supervision; Project administration; Writing—Review & Editing |
| 43 | Tùng Hữu Trịnh | Children’s Hospital 2, Ho Chi Minh City, Vietnam | Supervision; Project administration; Writing—Review & Editing |
| 44 | H Rogier van Doorn | Oxford University Clinical Research Unit Hanoi, Hanoi, Vietnam | Conceptualization; Funding acquisition; Project administration; Supervision; Validation; Writing—Original Draft |
|  |  | Centre for Tropical Medicine and Global Health, Nuffield Department of Medicine, University of Oxford, Oxford, UK |  |
| 45 | Paul Turner | Cambodia Oxford Medical Research Unit, Angkor Hospital for Children, Siem Reap, Cambodia | Conceptualization; Funding acquisition; Project administration; Supervision; Software; Validation; Formal analysis; Visualization; Writing—Original Draft |
|  |  | Centre for Tropical Medicine and Global Health, Nuffield Department of Medicine, University of Oxford, Oxford, UK |  |
| 46 | Sue J. Lee | Mahidol-Oxford Tropical Medicine Research Unit, Faculty of Tropical Medicine, Mahidol University, Bangkok, Thailand | Conceptualization; Supervision; Validation; Formal analysis; Writing—Original Draft |
|  |  | Centre for Tropical Medicine and Global Health, Nuffield Department of Medicine, University of Oxford, Oxford, UK |  |
|  |  | Department of Infectious Diseases, The Alfred Hospital and School of Translational Medicine, Monash University, Melbourne, Australia |  |

**Non-author contributors**

| Yoel Lubell | Centre for Tropical Medicine and Global Health, Nuffield Department of Medicine, University of Oxford, Oxford, UK | Conceptualization; Supervision |
| --- | --- | --- |
|  | Mahidol-Oxford Tropical Medicine Research Unit, Faculty of Tropical Medicine, Mahidol University, Bangkok, Thailand |  |
|  | Amsterdam Institute for Global Health and Development, Amsterdam, the Netherlands |  |
| Olivier Celhay | None | Software; Visualization |
| Vanapol Chamsukhee | Mahidol-Oxford Tropical Medicine Research Unit, Faculty of Tropical Medicine, Mahidol University, Bangkok, Thailand | Data Curation; Visualization |
| Prapass Wannapinij | Mahidol-Oxford Tropical Medicine Research Unit, Faculty of Tropical Medicine, Mahidol University, Bangkok, Thailand | Software; Data Curation |
| Sambou Bran | Cambodia Oxford Medical Research Unit, Angkor Hospital for Children, Siem Reap, Cambodia | Investigation |
| Chanpheaktra Ngoun | Cambodia Oxford Medical Research Unit, Angkor Hospital for Children, Siem Reap, Cambodia | Supervision; Project administration |
| Poda Sar | Cambodia Oxford Medical Research Unit, Angkor Hospital for Children, Siem Reap, Cambodia | Investigation |
| Antoinette A. A. Bediako-Bowan | Department of Surgery, University of Ghana Medical School, Accra, Ghana | Investigation |
| Appiah-Korang Labi | Department of Medical Microbiology, University of Ghana Medical School, Accra, Ghana | Investigation |
| Thomas Dankwah | Central Laboratory, Korle-Bu Teaching Hospital, Accra, Ghana | Investigation |
| Maud E. Fandoh | Department of Child Health, Korle-Bu Teaching Hospital, Accra, Ghana | Investigation |
| Margaret Gakpo | Department of Medicine, Korle-Bu Teaching Hospital, Accra, Ghana | Investigation |
| Esther Okine | Central Laboratory, Korle-Bu Teaching Hospital, Accra, Ghana | Investigation |
| Francis M. Tetteh | Pathology Department, 37 Military Hospital, Accra, Ghana | Investigation |
| Fitri Agustia Dewi | Oxford University Clinical Research Unit Indonesia, Faculty of Medicine Universitas Indonesia, Jakarta, Indonesia | Supervision |
| Michelle M Karman | Oxford University Clinical Research Unit Indonesia, Faculty of Medicine Universitas Indonesia, Jakarta, Indonesia | Supervision |
| Nunung Nuraini Mahpud | Oxford University Clinical Research Unit Indonesia, Faculty of Medicine Universitas Indonesia, Jakarta, Indonesia | Data curation |
| Theresa Valleri | Oxford University Clinical Research Unit Indonesia, Faculty of Medicine Universitas Indonesia, Jakarta, Indonesia | Data curation |
| Kevin Brevian | Pelni Hospital, Jakarta, Indonesia | Investigation |
|  | Faculty of Medicine, Krida Wacana Christian University, Jakarta, Indonesia |  |
| Erika Indrajaya | Pelni Hospital, Jakarta, Indonesia | Investigation |
|  | Faculty of Medicine, Atma Jaya Catholic University of Indonesia, Jakarta, Indonesia |  |
| Ikhlima Pramista Janaria | Pelni Hospital, Jakarta, Indonesia | Investigation |
|  | Persahabatan General Hospital, East Jakarta, Indonesia |  |
| Lili Hidayati Pangulu | Pelni Hospital, Jakarta, Indonesia | Investigation |
| Samuel Theodorus Sutanto | Pelni Hospital, Jakarta, Indonesia | Investigation |
|  | Faculty of Medicine, Krida Wacana Christian University, Jakarta, Indonesia |  |
| Martha Fitri Alextina Tatodi | Pelni Hospital, Jakarta, Indonesia | Investigation |
| Cliff Clarence Haliman | Department of Microbiology, Faculty of Medicine Universitas Indonesia, dr. Cipto Mangunkusumo National Hospital, Jakarta, Indonesia | Investigation |
| Anis Karuniawati | Department of Microbiology, Faculty of Medicine Universitas Indonesia, dr. Cipto Mangunkusumo National Hospital, Jakarta, Indonesia | Investigation |
| Aulia Khatib | Department of Child Health, Faculty of Medicine Universitas Indonesia, dr. Cipto Mangunkusumo National Hospital, Jakarta, Indonesia | Investigation |
| Djiwa Margono | Department of Microbiology, Faculty of Medicine Universitas Indonesia, dr. Cipto Mangunkusumo National Hospital, Jakarta, Indonesia | Investigation |
| Aghnia Nafila Tsaniy | Department of Child Health, Faculty of Medicine Universitas Indonesia, dr. Cipto Mangunkusumo National Hospital, Jakarta, Indonesia | Investigation |
|  | Kalideres Regional General Hospital, Jakarta, Indonesia |  |
| Agustin Indracahyani | University of Indonesia Hospital, Jakarta, Indonesia | Investigation |
|  | Department of Epidemiology, College of Public Heath, University of Iowa, USA |  |
| Iqbal Andri Makarim | University of Indonesia Hospital, Jakarta, Indonesia | Investigation |
| Ro Rabian Rein Roza | University of Indonesia Hospital, Jakarta, Indonesia | Investigation |
|  | Gadjah Mada University, Yogyakarta, Indonesia |  |
| Uci Rama Saputri | University of Indonesia Hospital, Jakarta, Indonesia | Investigation |
|  | Department of Anesthesiology, Faculty of Medicine Universitas Indonesia, dr. Cipto Mangunkusumo National Hospital, Jakarta, Indonesia |  |
| Adityo Susilo | Department of Internal Medicine, Faculty of Medicine Universitas Indonesia, dr. Cipto Mangunkusumo National Hospital, Jakarta, Indonesia | Investigation |
|  | University of Indonesia Hospital, Jakarta, Indonesia |  |
| Anne V. Amulele | KEMRI Wellcome Trust Research Programme, Kilifi, Kenya | Investigation |
| Jackline W. Gachoki | KEMRI Wellcome Trust Research Programme, Kilifi, Kenya | Supervision; Project administration |
| Wilson Gumbi | KEMRI Wellcome Trust Research Programme, Kilifi, Kenya | Investigation |
| Stephen N. Kamau | KEMRI Wellcome Trust Research Programme, Kilifi, Kenya | Data curation |
| Joyce W. Kigo | KEMRI Wellcome Trust Research Programme, Kilifi, Kenya | Data curation |
| Robert M. Musyimi | KEMRI Wellcome Trust Research Programme, Kilifi, Kenya | Supervision |
| Benedict C. Mvera | KEMRI Wellcome Trust Research Programme, Kilifi, Kenya | Supervision |
| Salim Y. Mwarumba | KEMRI Wellcome Trust Research Programme, Kilifi, Kenya | Supervision |
| Linnytiffan M. Ndumba | KEMRI Wellcome Trust Research Programme, Kilifi, Kenya | Investigation |
| Brian M. Nyamwaya | KEMRI Wellcome Trust Research Programme, Kilifi, Kenya | Supervision; Project administration |
| Rachel M. Inginia | Kitale County Referral Hospital, Kitale, Kenya | Investigation |
| James M. Njuguna | Kitale County Referral Hospital, Kitale, Kenya | Investigation |
| Anthony S. Katayi | Bungoma County Referral Hospital, Bungoma, Kenya | Investigation |
| Dickens I. Lubanga | Bungoma County Referral Hospital, Bungoma, Kenya | Investigation |
| Ko Chang | Mahosot Hospital, Vientiane, Lao PDR | Investigation |
| Vilada Chansamouth | Lao-Oxford-Mahosot Hospital-Wellcome Trust Research Unit, Microbiology Laboratory, Mahosot Hospital, Vientiane, Lao PDR | Supervision |
| Viengmon Davong | Microbiology Laboratory, Mahosot Hospital, Vientiane, Lao PDR | Supervision |
| Douangkham Phommachanh | Mahosot Hospital, Vientiane, Lao PDR | Investigation |
| Manoloth Sayarath | Mahosot Hospital, Vientiane, Lao PDR | Investigation |
| Keovongmany Senesouphonh | Mahosot Hospital, Vientiane, Lao PDR | Investigation |
| Peeyanout Thammavongsa | Lao-Oxford-Mahosot Hospital-Wellcome Trust Research Unit, Microbiology Laboratory, Mahosot Hospital, Vientiane, Lao PDR | Investigation |
| Khamphong Thanadabouth | Mahosot Hospital, Vientiane, Lao PDR | Investigation |
| Susath Vongphachanh | Mahosot Hospital, Vientiane, Lao PDR | Supervision |
| Manivanh Vongsouvath | Microbiology Laboratory, Mahosot Hospital, Vientiane, Lao PDR | Supervision |
| Khamloune Choumlivong | Setthathirath Hospital, Vientiane, Lao PDR | Investigation |
| Loungnilanh Manivanh | Microbiology Laboratory Setthathirath Hospital, Vientiane, Lao PDR | Investigation |
| Souphalack Sibounheuang | Setthathirath Hospital, Vientiane, Lao PDR | Investigation |
| Phoutmany Vongxay | Setthathirath Hospital, Vientiane, Lao PDR | Investigation |
| Sonesavanh Xayyalarth | Setthathirath Hospital, Vientiane, Lao PDR | Investigation |
| Patrick Musicha | Malawi Liverpool Wellcome Programme, Kamuzu University of Health Sciences, Blantyre, Malawi | Supervision |
| Sabina Dongol | Oxford University Clinical Research Unit Nepal, Lalitpur, Nepal | Supervision; Data Curation |
| Sushma Gyawali | Oxford University Clinical Research Unit Nepal, Lalitpur, Nepal | Supervision; Data Curation |
| Basudha Shrestha | phect-NEPAL/Kathmandu Model Hospital, Kathmandu, Nepal | Investigation |
| Basudha Khanal | BP Koirala Institute of Health Sciences, Dharan, Nepal | Investigation |
| Jola-Ade J Ajiboye | Department of Pharmaceutical Microbiology, University of Ibadan, Ibadan, Nigeria | Project administration |
| Veronica O Ogunleye | Department of Medical Microbiology and Parasitology, College of Medicine, University of Ibadan, Ibadan, Nigeria | Supervision |
| Faith Adekola | Department of Medical Microbiology and Parasitology, University College Hospital, Ibadan, Nigeria | Investigation |
| Abdulmumin Adewumi Aderinto | Department of Medical Microbiology and Parasitology, College of Medicine, University of Ibadan, Ibadan, Nigeria | Investigation |
| Fatima Adeyemi | Department of Medical Microbiology and Parasitology, University College Hospital, Ibadan, Nigeria | Investigation |
| Blessing Ajakaiye | Department of Medical Microbiology and Parasitology, College of Medicine, University of Ibadan, Ibadan, Nigeria | Investigation |
| Victoria Alonge | Department of Medical Microbiology and Parasitology, University College Hospital, Ibadan, Nigeria | Investigation |
| Folasade Bamidele | Department of Medical Microbiology and Parasitology, College of Medicine, University of Ibadan, Ibadan, Nigeria | Investigation |
| Benedicta Chike | Department of Medical Microbiology and Parasitology, College of Medicine, University of Ibadan, Ibadan, Nigeria | Investigation |
| Oluseyi Elaturoti | Department of Medical Microbiology and Parasitology, University College Hospital, Ibadan, Nigeria | Investigation |
| Oluwatobiloba S. Kazeem | Department of Medical Microbiology and Parasitology, College of Medicine, University of Ibadan, Ibadan, Nigeria | Investigation |
| Aderemi Oludiran Kehinde | Department of Medical Microbiology & Parasitology, College of Medicine, University of Ibadan, Ibadan, Nigeria | Investigation |
| Aishetu Nwankwo | Department of Medical Microbiology and Parasitology, University College Hospital, Ibadan, Nigeria | Investigation |
| Suliat Oladokun | Department of Medical Microbiology and Parasitology, College of Medicine, University of Ibadan, Ibadan, Nigeria | Supervision; Project administration |
| Regina E. Oladokun | College of Medicine, University of Ibadan and University College Hospital, Ibadan, Nigeria | Investigation |
| Chinyelo Ololo | Department of Medical Microbiology and Parasitology, University College Hospital, Ibadan, Nigeria | Investigation |
| Okainemen Precious Oluwalusi | Department of Medical Microbiology and Parasitology, College of Medicine, University of Ibadan, Ibadan, Nigeria | Supervision; Project administration |
| Olamide Omolawal | Department of Medical Microbiology and Parasitology, College of Medicine, University of Ibadan, Ibadan, Nigeria | Investigation |
| Oluwapelumi Mary Oni | Department of Medical Microbiology and Parasitology, College of Medicine, University of Ibadan, Ibadan, Nigeria | Investigation |
| Isaiah Adewale | Obafemi Awolowo University Teaching Hospitals Complex, Ile-Ife, Nigeria | Investigation |
| Anthony Anuforo | Obafemi Awolowo University Teaching Hospitals Complex, Ile-Ife, Nigeria | Investigation |
| Atinuke Ibrahim | Obafemi Awolowo University Teaching Hospitals Complex, Ile-Ife, Nigeria | Investigation |
| Taiwo S. Ogunbiyi | Obafemi Awolowo University Teaching Hospitals Complex, Ile-Ife, Nigeria | Investigation |
| Oluwafemi Akinyele Popoola | Department of Community Medicine, College of Medicine, University of Ibadan and University College Hospital, Ibadan, Nigeria | Investigation |
| Thomas Kesteman | Oxford University Clinical Research Unit Hanoi, Hanoi, Vietnam | Investigation |
| Nguyen Phuong Mai | Oxford University Clinical Research Unit Hanoi, Hanoi, Vietnam | Supervision; Data Curation |
| Nguyen Quynh Mai | Oxford University Clinical Research Unit Hanoi, Hanoi, Vietnam | Supervision; Data Curation |
| Nguyen Thi Tam | Oxford University Clinical Research Unit Hanoi, Hanoi, Vietnam | Investigation |
| Nguyen Thi Hong Thuong | Oxford University Clinical Research Unit Hanoi, Hanoi, Vietnam | Investigation |
| Agilakumari Pragasam | Oxford University Clinical Research Unit Hanoi, Hanoi, Vietnam | Investigation |
| Trinh Son Tung | Oxford University Clinical Research Unit Hanoi, Hanoi, Vietnam | Investigation |
| Vu Thi Ngoc Bich | Oxford University Clinical Research Unit Hanoi, Hanoi, Vietnam | Investigation |
| Vu Tien Viet Dung | Oxford University Clinical Research Unit Hanoi, Hanoi, Vietnam | Supervision; Data Curation |
| Dao Thanh Hai | National Hospital of Tropical Diseases, Hanoi, Vietnam | Investigation |
| Đoàn Duy Thành | National Hospital of Tropical Diseases, Hanoi, Vietnam | Investigation |
| Dong Phu Khiem | National Hospital of Tropical Diseases, Hanoi, Vietnam | Investigation |
| Hoàng Thùy Trang | National Hospital of Tropical Diseases, Hanoi, Vietnam | Investigation |
| Lương Hương Giang | National Hospital of Tropical Diseases, Hanoi, Vietnam | Investigation |
| Nguyen Quoc Phuong | National Hospital of Tropical Diseases, Hanoi, Vietnam | Investigation |
|  | Hanoi Medical University, Hanoi, Vietnam |  |
| Nguyen Thi Ha | National Hospital of Tropical Diseases, Hanoi, Vietnam | Investigation |
| Ninh Thi Ngoc | National Hospital of Tropical Diseases, Hanoi, Vietnam | Investigation |
| Pham Thi Dung | National Hospital of Tropical Diseases, Hanoi, Vietnam | Investigation |
| Phan Manh Cuong | National Hospital of Tropical Diseases, Hanoi, Vietnam | Investigation |
| Tran Van Bac | National Hospital of Tropical Diseases, Hanoi, Vietnam | Investigation |
| Van Dinh Trang | National Hospital of Tropical Diseases, Hanoi, Vietnam | Investigation |
| Dương Thái Duy | Hue Central Hospital, Hue, Vietnam | Investigation |
| Hoàng Văn Long | Hue Central Hospital, Hue, Vietnam | Investigation |
| Lê Thị Thu | Hue Central Hospital, Hue, Vietnam | Investigation |
| Mai Văn Tuấn | Hue Central Hospital, Hue, Vietnam | Investigation |
| Nguyễn Tất Dũng | Hue Central Hospital, Hue, Vietnam | Investigation |
| Nguyễn Thành Đạt | Hue Central Hospital, Hue, Vietnam | Investigation |
| Nguyễn Xuân Hiền | Hue Central Hospital, Hue, Vietnam | Investigation |
| Nguyễn Thị Bạch Oanh | Hue Central Hospital, Hue, Vietnam | Investigation |
| Nguyễn Thị Diệu Đoan | Hue Central Hospital, Hue, Vietnam | Investigation |
| Nguyễn Thị Hương | Hue Central Hospital, Hue, Vietnam | Investigation |
| Nguyễn Thị Yến Lan | Hue Central Hospital, Hue, Vietnam | Supervision |
| Phan Lê Quỳnh Thi | Hue Central Hospital, Hue, Vietnam | Investigation |
| Phan Thị Phương | Hue Central Hospital, Hue, Vietnam | Investigation |
| Trần Văn Bình | Hue Central Hospital, Hue, Vietnam | Investigation |
| Trần Quang Nhật | Hue Central Hospital, Hue, Vietnam | Investigation |
| Trần Đức Huy | Hue Central Hospital, Hue, Vietnam | Investigation |
| Trần Thị Huyền Trân | Hue Central Hospital, Hue, Vietnam | Investigation |
| Bùi Thế Trung | Children’s Hospital 2, Ho Chi Minh City, Vietnam | Investigation |
| Hồ Kiều Giang | Children’s Hospital 2, Ho Chi Minh City, Vietnam | Investigation |
| Hồ Thị Minh Thảo | Children’s Hospital 2, Ho Chi Minh City, Vietnam | Investigation |
| Lê Ngọc Ánh | Children’s Hospital 2, Ho Chi Minh City, Vietnam | Investigation |
| Lê Thị Hồng Hạnh | Children’s Hospital 2, Ho Chi Minh City, Vietnam | Investigation |
| Nguyễn Minh Ngọc | Children’s Hospital 2, Ho Chi Minh City, Vietnam | Supervision |
| Nguyễn Hà Đức | Children’s Hospital 2, Ho Chi Minh City, Vietnam | Supervision |
| Nguyễn Thanh Thiện | Children’s Hospital 2, Ho Chi Minh City, Vietnam | Investigation |
| Nguyễn Cao Minh Uyên | Children’s Hospital 2, Ho Chi Minh City, Vietnam | Investigation |
| Nguyễn Thị Quỳnh Anh | Children’s Hospital 2, Ho Chi Minh City, Vietnam | Supervision |
| Nguyễn Thị Kim Nhi | Children’s Hospital 2, Ho Chi Minh City, Vietnam | Investigation |
| Nguyễn Thị Kim Anh | Children’s Hospital 2, Ho Chi Minh City, Vietnam | Investigation |
| Nguyễn Thị Diệu Trường | Children’s Hospital 2, Ho Chi Minh City, Vietnam | Investigation |
| Nguyễn Thị Nhiều | Children’s Hospital 2, Ho Chi Minh City, Vietnam | Investigation |
| Nguyễn Thị Lan Phương | Children’s Hospital 2, Ho Chi Minh City, Vietnam | Investigation |
| Nguyễn Thị Thùy Trang | Children’s Hospital 2, Ho Chi Minh City, Vietnam | Investigation |
| Phạm Thị Đức Lợi | Children’s Hospital 2, Ho Chi Minh City, Vietnam | Investigation |
| Trần Thị Bích Kim | Children’s Hospital 2, Ho Chi Minh City, Vietnam | Investigation |
| Trịnh Thị Hồng Phương | Children’s Hospital 2, Ho Chi Minh City, Vietnam | Investigation |
